# Engineering cascade biocatalysis in whole cells for syringic acid bioproduction

**DOI:** 10.1186/s12934-024-02441-x

**Published:** 2024-06-01

**Authors:** Xin Liu, Yi An, Haijun Gao

**Affiliations:** School of Life Science, Beijing Institute of Technology, No 5 Zhongguancun South Street, Haidian District, Beijing, 100081 China

**Keywords:** Syringic acid, Enzyme cascade reaction, Whole cells catalysis, Methyltransferase, Shikimic acid, NADPH

## Abstract

**Background:**

Syringic acid (SA) is a high-value natural compound with diverse biological activities and wide applications, commonly found in fruits, vegetables, and herbs. SA is primarily produced through chemical synthesis, nonetheless, these chemical methods have many drawbacks, such as considerable equipment requirements, harsh reaction conditions, expensive catalysts, and numerous by-products. Therefore, in this study, a novel biotransformation route for SA production was designed and developed by using engineered whole cells.

**Results:**

An *O*-methyltransferase from *Desulfuromonas acetoxidans* (*DesA*OMT), which preferentially catalyzes a methyl transfer reaction on the meta-hydroxyl group of catechol analogues, was identified. The whole cells expressing *Des*AOMT can transform gallic acid (GA) into SA when *S*-adenosyl methionine (SAM) is used as a methyl donor. We constructed a multi-enzyme cascade reaction in *Escherichia coli*, containing an endogenous shikimate kinase (AroL) and a chorismate lyase (UbiC), along with a *p*-hydroxybenzoate hydroxylase mutant (PobA^**^) from *Pseudomonas fluorescens*, and *DesA*OMT; SA was biosynthesized from shikimic acid (SHA) by using whole cells catalysis. The metabolic system of chassis cells also affected the efficiency of SA biosynthesis, blocking the chorismate metabolism pathway improved SA production. When the supply of the cofactor NADPH was optimized, the titer of SA reached 133 μM (26.2 mg/L).

**Conclusion:**

Overall, we designed a multi-enzyme cascade in *E. coli* for SA biosynthesis by using resting or growing whole cells. This work identified an *O*-methyltransferase (*Des*AOMT), which can catalyze the methylation of GA to produce SA. The multi-enzyme cascade containing four enzymes expressed in an engineered *E. coli* for synthesizing of SA from SHA. The metabolic system of the strain and biotransformation conditions influenced catalytic efficiency. This study provides a new green route for SA biosynthesis.

**Graphical Abstract:**

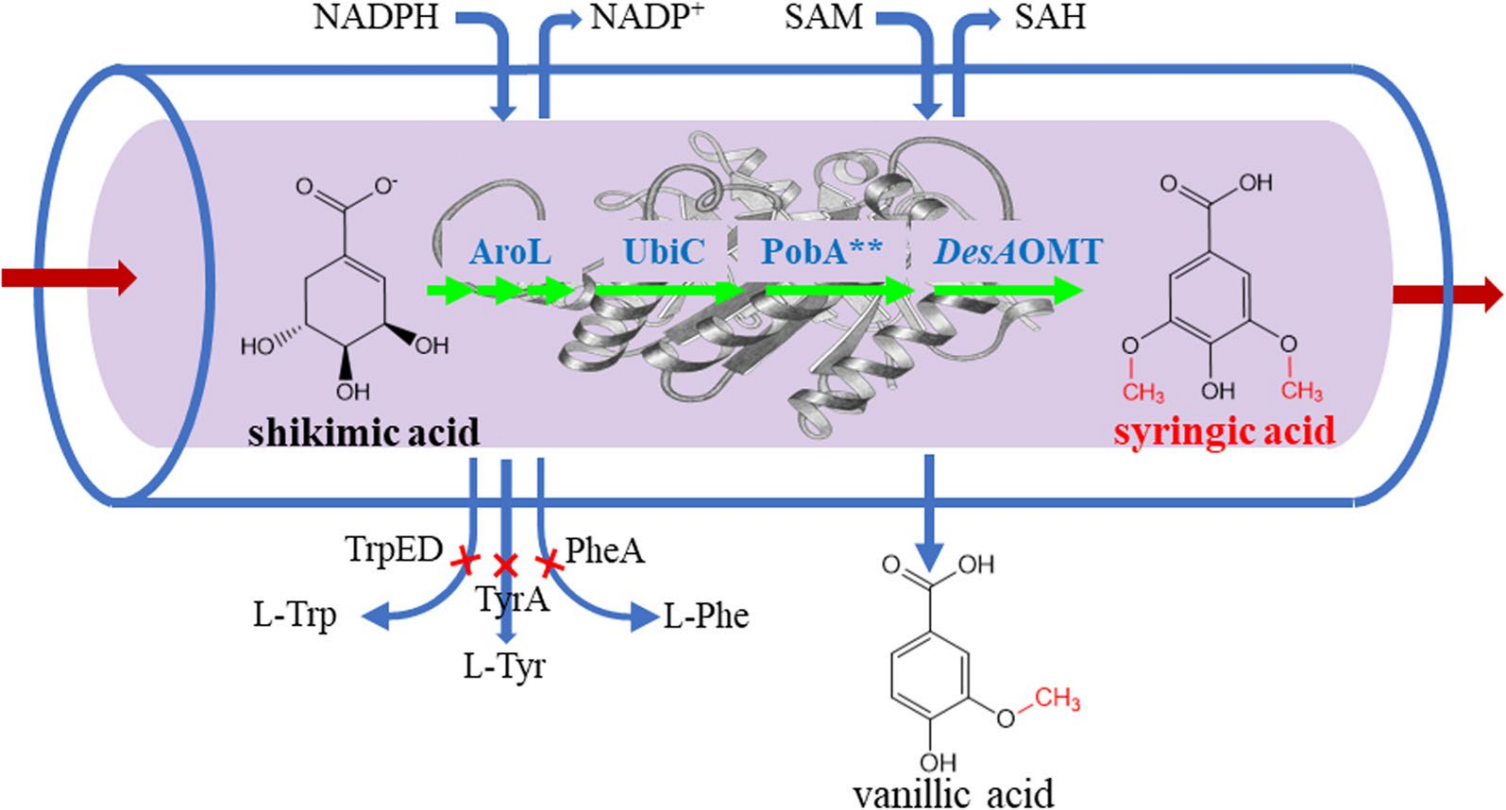

**Supplementary Information:**

The online version contains supplementary material available at 10.1186/s12934-024-02441-x.

## Introduction

Plant natural products (PNPs) exhibit a wide range of physiological functions, such as antioxidant [1], antitumor [2], and anti-inflammatory [3] effects. Therefore, they are important sources of drugs, cosmetics, and additives, and have extremely high application value. PNPs are mainly extracted from plants, however, this traditional manufacturing approach has many disadvantages, such as a long plant growth period, low natural product content, complicated purification processes, and severe damage to biological resources [4]. Some PNPs can also be produced by chemical synthesis. Due to the complex structure of most natural products with multiple chiral centers, structural analogs are formed during chemical processing, which makes subsequent separation and purification difficult [5]. Moreover, owing to complicated processes, low conversion rates, high energy consumption, and environmental unfriendliness, chemical synthesis of PNPs does not easily meet the needs of industrial processes [6]. In contrast, based on the principles of synthetic biology, engineered cells are designed and constructed for the continuous and efficient production of PNPs, representing a green and efficient approach for the preparation of natural products, reducing or eliminating the drawbacks of chemical synthesis. Currently, biological approaches are used to manufacture many important plant natural products such as morphine [7], artemisinic acid [8], ginsenosides [9], and anthocyanins [10].

Syringic acid (4-hydroxy-3,5-dimethoxybenzoic acid, SA) is a natural phenolic compound commonly found in fruits, vegetables, and herbs. Due to the presence of methoxy groups at the 3 and 5 positions of the aromatic ring, SA has strong antioxidant [11], antibacterial [12], anti-inflammatory [13], and hepatic and neuroprotective activities [14], leading to a wide range of therapeutic applications in the prevention of diabetes [15], cardiovascular disease [16], cancer [17], etc. 2-Ethylhexyl syringate, a derivative of SA, has been used as a component of dental adhesives due to its tensile strength, water solubility, and anticaries properties [18]. SA plays a role in the communication between plants and soil microorganisms by altering the microbial community structure of the soil rhizosphere and inhibiting the growth of cucumber plants [19]. SA can also promote the microbial degradation of herbicides in the soil-plants system, showing bioremediation potential [20].

SA is produced mainly by chemical synthesis, in which trimethyl ether gallate (3,4,5-trimethoxybenzoic acid) is used as a raw material, after which the methyl group at the 4-position is removed under alkaline or acidic conditions [21]. In addition, syringaldehyde can also be used as a raw material for the production of SA via multiple steps including esterification with acetic anhydride, oxidation with hydrogen peroxide, and hydrolysis under alkali/acid conditions [22]. However, there are many shortcomings in these chemical approaches, such as considerable equipment requirements, harsh reaction conditions, expensive catalysts, and many byproducts.

Reconstructing metabolic pathways is an important strategy for the metabolic engineering of natural product synthesis. Studies have shown that SA in plants is synthesized via a series of enzymatic reactions including the shikimate pathway [23], however, this route is too complex to either directly modify plants to enhance SA production or transplant the pathway components into microorganisms. The shikimate pathway is the main bridge between carbohydrate metabolism and aromatic chemicals, and modification of the shikimate pathway in microorganisms results in overproduction of a variety of high value-added metabolites [24]. Gallic acid (GA) was biochemically synthesized by using 3-dehydroshikimic acid (DHS), an intermediate of the shikimate pathway, as a precursor, thereby expanding the shikimate pathway to GA and overcoming the restrictions of isolating GA from scarce natural sources [25]. GA can also be synthesized via enzymatic catalytic hydroxylation of 3,4-dihydroxybenzoic acid (3,4-DHBA) by a *p*- hydroxybenzoate hydroxylase mutant (PobA^**^) from *Pseudomonas aeruginosa*, thus establishing a promising pathway for the biosynthesis of GA and its derivatives [26]. It is speculated that SA biosynthesis was carried out by introducing two methoxy groups at the 3 and 5 positions of GA via an oxygen methylation reaction catalyzed by *O*-methyltransferases (OMTs). OMTs are widely found in plants [27], animals [28], and microorganisms [29, 30] and usually use S-adenosyl methionine (SAM) as a methyl donor to catalyze methylation reactions in organisms.

**Fig. 1:**
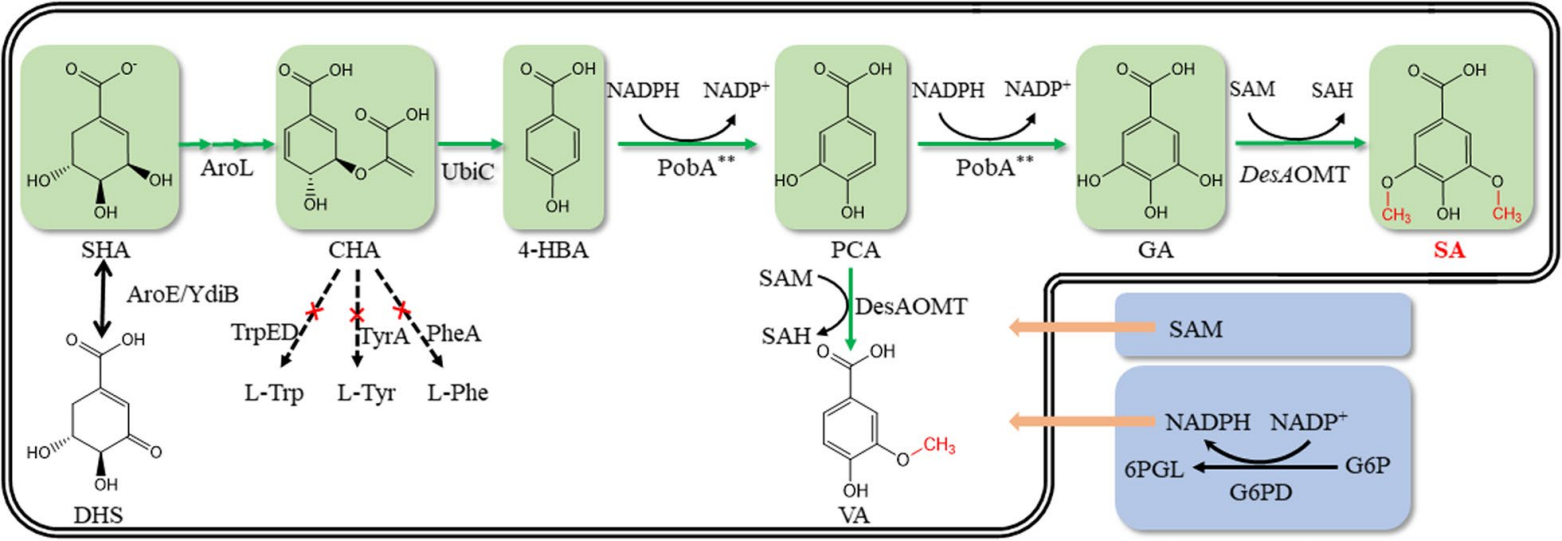
Multienzyme cascade reaction for the synthesis of SA from SHA. The cascade reactions designed in this work are represented by green arrows. The multiple arrows in succession illustrate multiple-step reactions. The blue arrow indicates the byproduct of the cascade reaction. Red crosses indicate the disruption of the corresponding genes. Abbreviations: DHS, 3-dehydroshikimic acid; SHA, shikimic acid; CHA, chorismic acid; 4-HBA, hydroxybenzoic acid; PCA, protocatechuic acid; GA, gallic acid; VA, vanillic acid; SA, syringic acid; G6P, glucose-6-phosphate; 6PGL, 6-phosphogluconolactone. AroE, shikimate dehydrogenase; YdiB, quinate/shikimate dehydrogenase; AroL, shikimate kinase; UbiC, chorismate lyase; PobA^**^, *p*-hydroxybenzoate hydroxylase mutant (Y385F/T294A); *DesA*OMT, *O*-methyltransferase from *Desulfuromonas acetoxidans*; G6PD, glucose-6-phosphate dehydrogenase

To test the hypothesis proposed above, a multienzyme cascade reaction was designed for SA biosynthesis catalyzed by enzymes from *E. coli* and other microorganisms (Fig. 1). The cascade reaction started from the conversion of shikimic acid (SHA), which might be produced by microbial fermentation, to chorismate catalyzed by an endogenous shikimate kinase (AroL) and other native enzymes, after which the chorismate was transformed to 4-hydroxybenzoic acid (4-HBA) catalyzed by a chorismate lyase (UbiC). Next, a reaction catalyzed by Y385F/T294A PobA** (a PobA mutant) added two hydroxyl groups to 4-HBA at the 3 and 5 positions to form GA. Due to the structural similarity between GA and catechol, a catechol-*O*-methyltransferase (COMT), which uses catechol as a primary substrate, was identified and employed to produce SA from GA. This multienzyme cascade catalytic reaction was constructed in *E. coli* for SA biosynthesis, and the metabolic network of the host strain and catalytic process were optimized to increase the yield of SA. This study provides a new biotransformation route for SA production and demonstrates the feasibility of bioproducting SA by engineering microorganisms.

## Materials and methods

### Chemicals and reagents

Takaro (Dalian, China) and New England Biolabs (Beijing, China) provided enzymes and kits for molecular cloning experiments. For the cultivation process, Oxoid (Basingstoke, UK) supplied tryptone and yeast extract. Oligonucleotides were synthesized by Sangon Biotech (Shanghai, China). All remaining reagents and chemicals utilized in the work were purchased from Sigma-Aldrich (Shanghai, China).

### Construction of plasmids and strains

The strains, plasmids, and primers used in this study are provided in supplementary materials. *E. coli* DH5α and *E. coli* BL21(DE3) were used for plasmid construction and single enzyme catalysis, and *E. coli* MG1655 was used as a host strain for the expression of the multienzyme cascade. The plasmid pET-28a was used for screening OMTs, and the plasmid pTrc99a was used to express multienzyme cascade. The genes encoding eleven OMTs, and the gene encoding PobA^**^ were synthesized by Sangon Biotech (Shanghai, China). The genes *aroL* and *ubiC* were amplified using *E. coli* MG1655 genomic DNA as a template.

To achieve multienzyme cascade catalytic synthesis of SA, the genes coding for four enzymes were ligated by overlapping extension PCR [31] and inserted into pTrc99a by Gibson assembly [32, 33]. Knockout of *aroE*, *ydiB*, *trpED*, *pheA*, and *tyrA* genes was performed using the CRISPR-Cas gene editing system [34], in which the plasmids pCas and pTargetF were used. The online tool CHOPCHOP (https://chopchop.cbu.uib.no/), was used to select the sgRNA sequences of the target genes. The upstream and downstream homologous DNA fragments (500 bp) and sgRNAs were amplified via PCR and inserted into the pTargetF vector to generate the plasmids pTarget-*aro*E, pTarget-*ydi*B, pTarget-*trpED*, and pTarget-*phe*A-*tyr*A.

### Cultivation conditions

Lysogeny broth (LB) medium, which consisted of 5 g/L yeast extract, 10 g/L peptone, and 10 g/L sodium chloride, and supplemented with appropriate ampicillin or kanamycin sulfate, was used for pre-culture and culture. A single colony was inoculated into 4 mL of LB medium and incubated in a rotating shaker (Zhicheng Inc., Shanghai, China) at 190 r/min and 37 °C. Five hundred microlitres of each culture were transferred to a 250 mL flask containing 50 mL of LB medium and incubated at 37 ℃ for 5 h. Afterward, isopropyl-β-d-thiogalactoside (IPTG) was added to a final concentration of 0.5 mM for induction. The cultures were then grown at 37 °C (for the *E. coli* strain containing pTrc99a derivatives) or 30 °C (for the *E. coli* strain containing pET-28a derivatives) for an additional 16 h.

### Biotransformation using resting whole cells

The bacterial culture mixture was centrifuged at 4 °C and 4000 rpm for 10 min to collect the cells, after which the cells were washed and resuspended in 50 mM phosphate-buffered saline (PBS, pH 7.2–7.4). The reaction was carried out in a 24-deep well plate, each well containing a 1 mL mixture composed of the whole cells (OD_600nm_ of 9), various concentrations of substrate, 2.5 mM SAM, 1 mM MgCl_2_, and 50 mM PBS buffer (pH 7.2–7.4), and was stirred in a rotating shaker (Zhicheng Inc, Shanghai, China) at 37 °C and 190 rpm for 8 h. An NADPH regeneration mixture (iPhase Biosciences, China) was added to the reaction system when needed.

### Biotransformation using growing whole cells

The engineered *E. coli* strain was cultivated in shake flasks at 190 rpm and 37 °C for 5 h. OMT gene expression was induced by adding 0.5 mM IPTG. Additionally, various concentrations of substrate, 2 mM SAM, 1 mM MgCl2, and 2 g/L glucose were added to the culture medium. The cultivation was then continued at 37 °C for 24 h to perform biotransformation.

### Analytic methods

Cell growth was determined by measuring the OD_600nm_ using a UVmini-1240 spectrophotometer (Shimadzu Corporation, Kyoto, Japan). SA, intermediates, and byproducts in the reaction mixture were analyzed using high-performance liquid chromatography (HPLC). Briefly, the samples were centrifuged at 12,000 rpm for 2 min, after which the supernatant was collected, filtered through 0.22 μm filters, and subsequently injected into an HPLC system (Agilent 1260 Infinity II, Santa Clara, USA) equipped with a GL Science InertSustain C-18 column (4.6 × 250 mm). The mobile phase was a mixture of water containing 0.1% acetic acid and acetonitrile (80:20, V/V) and was used at a flow rate of 0.8 mL/min. The temperature of the column was maintained at 32 °C throughout the process of analysis. A 10 μL portion of each sample was injected for analysis using an automated injector. An ultraviolet detector was used to analyze the samples at a wavelength of 275 nm. The chemical concentrations were estimated by comparing the peak areas and retention times of the chemicals with those of the standard curves generated with reference substances.

Other phenolic compounds and corresponding oxymethyl compounds involved in this study were also analyzed by using HPLC, and the specific conditions are provided in Supplementary Material.

## Results

### Screening of *O*-methyltransferases

**Fig. 2:**
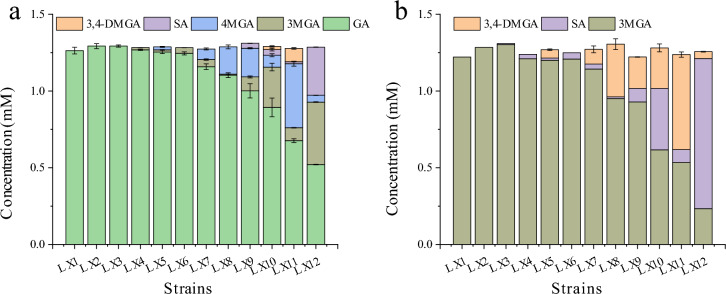
Catalytic activities of various OMTs expressed in *E. coli* BL21. **a** Resting whole cells catalysis using GA as substrate. **b** Resting whole cells catalysis using 3MGA as a substrate. All the *E. coli* strains were cultivated in shake flasks at 190 rpm and 37 °C for 5 h, 0.5 mM IPTG was added to induce gene expression; and subsequent cultivation was conducted at 30 °C for 18 h. Afterward, the cells were collected. Resting cells (OD_600nm_ of 9) were subjected to catalysis of 1.2 mM GA or 3MGA for 8 h. The following strains (expressing OMTs) were used: LX1 (control strain), LX2 (*StiA*OMT from *Stigmatella aurantiaca*), LX3 (*StyL*OMT from *Stylonychia lemnae*), LX4 (*KibP*OMT from *Kibdelosporangium phytohabitans*), LX5 (*Syn*OMT from *Synechocystis sp.*), LX6 (*RetF*OMT, *Reticulomyxa filosa*), LX7 (*OmnB*OMT from *Omnitrophica bacterium*), LX8 (*MyxX*OMT from *Myxococcus xanthus*), LX9 (*MycT*OMT from *Mycobacterium tuberculosis*), LX10 (*StrA*OMT from *Streptomyces avermitilis*), LX11 (*PhoA*OMT from *Phormidium ambiguum*), and LX12 (*DesA*OMT from *Desulfuromonas acetoxidans*). The error bars represent the standard deviations of the means of three independent measurements

To perform the enzyme-catalyzed synthesis of SA from GA, an *O*-methyltransferase that catalyzes multiple *O*-methylations of GA is needed. To screen the OMTs that catalyzed the methylation of the meta-hydroxyl group of GA molecules, the genes encoding the various OMTs listed in Table S4 were inserted into the pET-28a vector, and the resulting strains were cultivated to test their characteristics. The whole cells expressing the OMTs were used for GA and 3-*O*-methyl GA (3MGA) catalysis for 8 h, and the results are shown in Fig. 2. The strain LX12 expressing *DesA*OMT had the highest catalytic activity toward GA (Fig. 2a); 1.2 mM GA was converted to 0.41 mM 3MGA, 0.04 mM 4-*O*-methyl GA (4MGA) and 0.31 mM SA, and the yield of SA reached 24.2%. Figure 2b shows the whole cells catalytic activity of 11 OMTs on 3MGA. Similarly, the strain LX12 also had the highest catalytic activity on 3MGA; 1.2 mM 3MGA was converted into 0.98 mM SA and 0.04 mM 5-hydroxy-3,4-dioxymethylbenzoic acid (3,4-DMGA), and the yield of SA reached 78.4%. Therefore, *DesA*OMT, which had greater activity on GA in the OMTs listed above, was selected for follow-up experiments.

### Substrate specificity and regiospecificity of DesAOMT

To understand the catalytic properties of *DesA*OMT, the substrate specificity and regiospecificity of *DesA*OMT were assessed using whole cells catalysis of various phenolic compounds; the results are shown in Table 1. Monohydroxy phenolic acids could not be used as substrates. For phenolic acid compounds with two hydroxyl groups, *DesA*OMT preferred the positions (3, 4) and (3, 5) rather than (2, 5) and (2, 6). Surprisingly, although the enzyme could catalyze the methylation reaction of 3, 4-dihydroxybenzoic acid (protocatechuic acid) and 3,4-dihydroxycinnamic acid (caffeic acid) but not 3,4-dihydroxyphenylacetic acid, all the substituent groups, including the carboxyl and hydroxyl groups attached to the aromatic ring, seem to contribute to the substrate selectivity and regiospecificity of *DesA*OMT, this is the result of interactions among multiple factors, including not only specific amino acids in the enzyme, but also the substrate molecular structure and characteristics [29] and reaction conditions [35, 36], and may not be limited by these factors. The interaction between *DesA*OMT and GA was also studied using Discovery Studio, the predicted *DesA*OMT-GA complex was shown in Fig. S2 that Lys87, Asp129, and Asn156 may contribute more to the catalytic activity of the enzyme.

**Table 1:** Substrate selectivity and regioselectivity of *DesA*OMT

Substrate	Structure	Relative activity	Meta/para ratio
2-hydroxybenzoic acid (2HBA)		0	–
3-hydroxybenzoic acid (3HBA)		0	–
4-hydroxybenzoic acid (4HBA)		0	–
2,5-dihydroxybenzoic acid (2,5-DHBA)		0	–
2,6-dihydroxybenzoic acid (2,6-DHBA)		0	–
3,4-dihydroxybenzoic acid (3,4-DHBA)		41.8	0.065
3,5-dihydroxybenzoic acid (3,5-DHBA)		30.4	–
3,4-dihydroxyphenylacetic acid (3,4-DHAA)		0	–
3,4-Dihydroxycinnamic acid (Caffeic Acid)		13.9	2.67
Gallic acid (GA)		100	18.75

*E. coli* strain LX12 was used to examine the substrate selectivity and regioselectivity of *DesA*OMT. The strain was cultivated in shake flasks at 190 rpm and 37 °C for 5 h, 0.5 mM IPTG was added to induce gene expression; and subsequent cultivation was conducted at 30 °C for 18 h. Then the cells were collected (OD_600nm_ of 9) and subjected to catalysis with various substrates at the concentration of 1.2 mM for 8 h

### Whole cells catalytic synthesis of SA from GA

Using GA as a substrate, resting whole cells were employed to synthesize SA. The *E. coli* strain LX12 was cultivated in shake flasks at 190 rpm and 37 °C for 5 h, 0.5 mM IPTG was added to induce gene expression, and subsequent cultivation was conducted at 30 °C for 18 h. Then, the cells were collected (OD_600nm_ of 9) and subjected to catalysis of GA for 8 h.

**Fig. 3:**
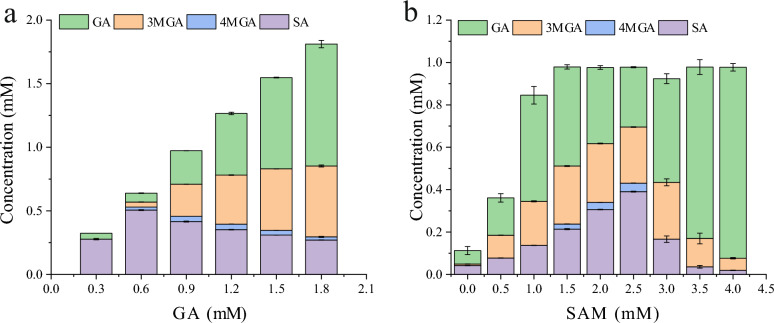
Effects of the concentration of the substrate GA (**a**) and cosubstrate SAM (**b**) on the whole-cell catalytic synthesis of SA from GA. The *E. coli* strain LX12 was cultivated in shake flasks at 190 rpm and 37 °C for 5 h, 0.5 mM IPTG was added to induce gene expression, and subsequent cultivation was conducted at 30 °C for 18 h. Then, the cells were collected. The resting cells (OD_600nm_ of 9) were subjected to catalysis of GA for 8 h. The error bars represent the standard deviations of the means of three independent measurements

The effects of the substrate GA concentration on the SA synthesis were investigated (Fig. 3a). The conversion of GA gradually decreased with the increasing GA concentration, while the amount of byproducts such as 3MGA progressively increased. This implied that the mechanism of the bioconversion of SA is random methylation of hydroxyl groups, rather than the simultaneous methylation of two hydroxyl groups.

*S*-Adenosylmethionine (SAM) is a methyl donor used in most methylation processes catalyzed by *O*-methyltransferases; after donating its methyl, SAM is converted to the neutral compound *S*-adenosylhomocysteine (SAH). The effect of SAM concentration on the catalytic activity of *DesA*OMT was investigated (Fig. 3b), in which 1.0 mM GA was used in the substrate mixture. The yield of SA increased along with the increasing of SAM concentration from 0–2.5 mM, while it decreased followed the decreasing trend of SAM concentration from 2.5–4.0 mM. These results indicated that low SAM concentration promoted SA synthesis, whereas high SAM concentration inhibited SA production.

In addition, the effects of other biotransformation conditions on SA synthesis were also examined (Fig. S1). The concentration of Mg^2+^ in the range of 0–6 mM had no effect on the yield of SA; high concentration of SA in the range of 0–0.9 mM did not inhibit the reaction; glucose addition in the range of 0–3 mM did not promote SA synthesis significantly; and the surfactant CTAB in the range of 0–0.9 mM and lysozyme in the range of 0–1.5 mg/mL did not increase the yield of SA.

### Bioconversion of SHA to SA

**Fig. 4:**
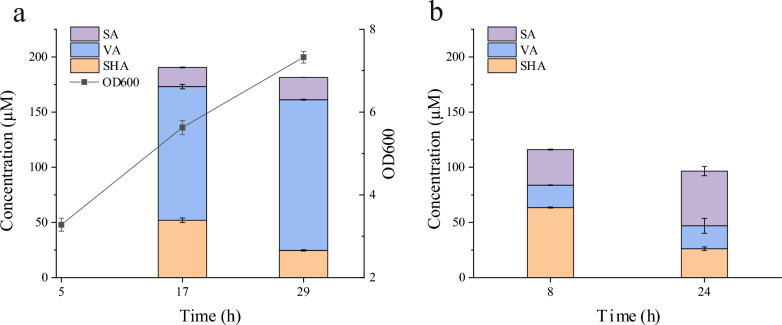
Bioconversion of SHA to SA. **a** The biosynthesis of SA by using growing whole cells. *E. coli* LX15 was cultivated in shake flasks at 190 rpm and 37 °C for 5 h, 0.5 mM IPTG was added to induce gene expression, a substrate mixture containing 2 mM SHA was added, and subsequent cultivation was conducted at 37 °C for 24 h. **b** The biosynthesis of SA by using resting whole cells. *E. coli* LX15 was cultivated in shake flasks at 190 rpm and 37 °C for 5 h, 0.5 mM IPTG was added to induce gene expression, and subsequent cultivation was conducted at 37 °C for 18 h. Then, the cells were collected (OD_600nm_ of 9) and subjected to catalysis of 2 mM SHA for 24 h. The error bars represent the standard deviations of the means of three independent measurements

SHA is a metabolite of the shikimate pathway, and its efficient biosynthesis has been accomplished by modifying the metabolic network of *E. coli* [37]. To use SHA as a precursor in the biosynthesis of SA, the plasmid pT-AUPD was constructed, containing the genes *aroL*, encoding the shikimate kinase, and *ubiC*, encoding the chorismate lyase from *E. coli*; pobA^**^, encoding a *p*-hydroxybenzoate hydroxylase mutant from *P. fluorescens*, and Dace_1119, encoding the *DesA*OMT; this plasmid was introduced into *E. coli* DH5α, resulting in the *E. c*oli strain LX15. We used both growing and resting whole cells for SA production from SHA. The engineered *E. coli* strain LX15 was cultivated in shake flasks at 190 rpm and 37 °C for 5 h, we induced *DesA*OMT expression by adding 0.5 mM IPTG. For growing cell biotransformation, a substrate mixture containing 2 mM SHA was added to the culture medium, and subsequent cultivation was conducted at 37 °C for 24 h to perform biotransformation. For resting whole cell biotransformation, cultivation was conducted at 37 °C for 18 h after induction of *DesA*OMT expression, then the cells were collected (OD_600nm_ of 9), and subjected to catalysis of 2 mM SHA at 37 °C for 24 h. As illustrated in Fig. 4a, after catalyzing by growing *E. coli* LX15 cells for 24 h, the concentrations of SA and vanillic acid (VA), a byproduct, reached 20 μM (4.0 mg/L) and 136 μM (22.7 mg/L), respectively, when 2 mM SHA was used as substrate. By contrast, resting *E. coli* LX15 cells were able to convert 2 mM SHA to 50 μM (9.9 mg/L) SA and 21 μM (3.5 mg/L) VA within 24 h, resulting in a yield of 3.6% (Fig. 4b). This yield also indicated that most SHA in the reaction mixture were metabolized or utilized via the metabolic network of the host cell.

### Modification of the metabolic network of the strain to promote SA synthesis

**Fig. 5:**
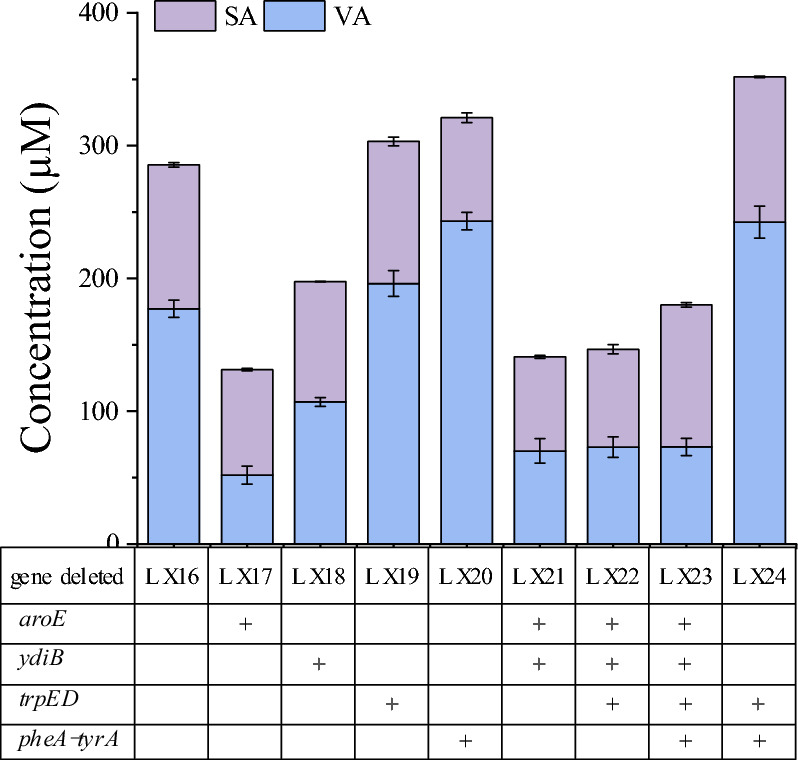
Effects of metabolic network modification of the *E. coli* strain on the yields of SA and VA. The *E. coli* strains tested were cultivated in shake flasks at 190 rpm and 37 °C for 5 h, 0.5 mM IPTG was added to induce gene expression, and subsequent cultivation was conducted at 37 °C for 18 h. Then the cells were collected (OD_600nm_ of 9) and subjected to catalysis of 2 mM SHA for 8 h. The modified strains used were LX16 (control strain), LX17 (Δ*aroE*), LX18 (Δ*ydiB*), LX19 (Δ*trpED*), LX20 (Δ*phe*A-*tyr*A), LX21 (Δ*aroE* Δ*ydiB*), LX22 (Δ*aroE* Δ*ydiB* Δ*trpED*), LX23 (Δ*aroE* Δ*ydiB* Δ*trpED* Δ*pheA-tyrA*), and LX24 (Δ*trpED* Δ*pheA*-*tyrA*). The error bars represent the standard deviations of the means of three independent measurements

To further improve the bioconversion yield of SA from SHA by using whole cells biotransformation, the *E. coli* MG1655 metabolic network was modified to prevent SHA or the intermediates from being metabolized via the native metabolic network and allowing more C-flux into SA. The plasmid pT-AUPD was subsequently transformed into the modified strains, resulting in strains LX17–LX24. After cultivation and induction, resting whole cells were subjected to examination, and the results are shown in Fig. 5.

Compared with the unmodified original strain, strain LX19, with deleted *trpED* genes encoding anthranilate synthase components 1 and 2, and the strain LX20, with deleted *phe*A-*tyr*A genes encoding for prephenate dehydratase and prephenate dehydrogenase, respectively, exhibited increased VA production and slightly decreased SA production; strain LX24, with both *trpED* and *pheA*-*tyrA* deleted, exhibited increased production of both SA and VA, to 109 μM and 242 μM, respectively. Surprisingly, deletion of *aro*E or *ydi*B, which encodes for SHA dehydrogenase, resulted in decreased production of SA and VA, even after additional knockout of *trpED* or *phe*A-*tyr*A.

### Optimization of the NADPH supply to enhance SA synthesis

**Fig. 6:**
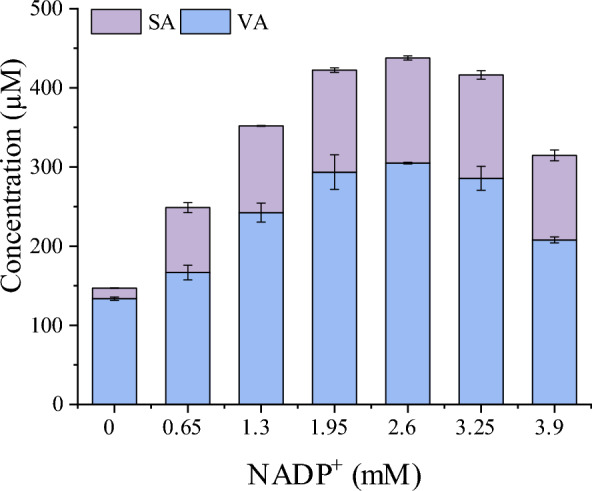
Effects of the NADPH supply in the reaction system on the yields of SA and VA. The *E. coli* strains tested were cultivated in shake flasks at 190 rpm and 37 °C for 5 h, 0.5 mM IPTG was added to induce gene expression, and subsequent cultivation was conducted at 37 °C for 18 h. Then the cells were collected (OD_600nm_ of 9) and subjected to catalysis of 2 mM SHA for 8 h. Strain LX24 was used to test the effects of the NADPH regeneration mixture supply, the NADP^+^ ranged from 0–3.9 mM in the reaction system. The error bars represent the standard deviations of the means of three independent measurements

NADPH is an essential cofactor for PobA in hydroxylation reactions. To investigate the effects of the NADPH supply on SA synthesis, NADPH was provided by adding a portion of in vitro NADPH regeneration mixture (glucose-6-phosphate dehydrogenase, glucose-6-phosphate, and NADP^+^) to the resting whole cells catalytic system, in which the strain LX24 was used; the results are shown in Fig. 6. The production of SA and VA was enhanced by the addition of the NADPH regeneration mixture. When the concentration of NADP^+^ added was in the range of 0–2.6 mM, the titers of SA ranged from 13.5 μM (2.7 mg/L) to 132.7 μM (26.2 mg/L), while the VA from 133.5 μM (22.4 mg/L) to 305 μM (51.3 mg/L). These results implied that NADPH levels were a critical factor in SA bioproduction, as the production of SA and VA were directly affected. However, the titers of SA and VA decreased when more NADPH regeneration mixture was added.

## Discussion

Methylation reactions catalyzed by methyltransferases are widespread in various organisms and are used to regulate many biological metabolic processes, such as cell signal transduction and the synthesis of complex metabolites. Depending on the target atom of methylation, methyltransferases are classified into oxygen-, carbon-, nitrogen-, sulfur-, and arsenic-directed types [38]. The substrates of methyltransferases include nucleotides, proteins, lipids, and small molecules. The most studied methyltransferase is OMT, which plays an important role in biological activities. In animals, OMTs are mainly used for the synthesis of catecholamine neurotransmitters and some hormones [39], and can also catalyze the methylation of flavonoids to reduce their carcinogenicity [40]. In plants, OMTs catalyze the synthesis of secondary metabolites, typically via the use of phenylpropanoid compounds, which contain hydroxyl groups, as substrates to regulate a variety of plant physiological processes [41].

With the development of structural biology techniques, an increasing number of crystal structures of OMTs have been elucidated. The catalytic triplet Lys-Asn-Asp has a very important role in catalytic activity of enzymes from plants. The same or similar catalytic residues are also present in microbial OMTs. A regioselective oxygen-methyltransferase (*DesA*OMT), which is capable of biocatalytic methylation at the 3- and 5-position hydroxyl groups of GA, was identified to produce SA using SAM as the methyl donor. The study on the interaction between *DesA*OMT and GA showed that the amino acid residues (Lys87, Asp129, and Asn156) (Fig. S2), which formed hydrogen bonds between *DesA*OMT and the substrate GA, are the same as those in the catalytic triplet of plant COMT, however, quite different from the active sites (Arg132, Asn156, Lys186) suggested by the sequence alignment of *DesA*OMT and SafC (PDB: 5LOG) [42]. Although the amino acid sequence of a protein determines its three-dimensional structure, protein folding is a complex process, and further evidence is needed to identify the active site of *DesA*OMT.

Sokolova et al. [42] found that *DesA*OMT methylated a diverse range of substrates, including catechol-like compounds such as flavonoids, coumarins, hydroxybenzoic acids, and their corresponding aldehydes, as well as non-catechol compounds like anthraquinone and indole. Unlike StrAOMT, *DesA*OMT exhibited lower regioselectivity, which remained consistent over a wide pH range. In this study, we also examined the substrates of *DesA*OMT, including protocatechuic acid, GA, and their derivative. In contrast to the findings of Sokolova et al. [42], our results showed that *DesA*OMT exhibited high regioselectivity, with a preference for the meta-position hydroxy group of Gallic acid and the para-position hydroxy group of 3,4-dihydroxybenzoic acid.

A sufficient supply of cofactors is a critical requirement for industrial biocatalysts processes. SAM is commonly used as a methyl donor in methylation reactions, but it is unstable [43] and expensive, and its demethylated product SAH inhibits the methylation reaction [44], thus limiting methyltransferase application. An in vivo SAM regeneration system was innovatively designed and developed to regenerate SAM through a six-enzyme cascade reaction, thus improving the production of methylated products [45]. An alternative SAM regeneration reaction was constructed in which methyl iodide was used as a stoichiometric methyl donor to regenerate SAM via catalysis by halide methyltransferase; this efficient methyl supply system was successfully applied to reactions catalyzed by *N*- and *O*-methyltransferases [46]. Betaine-homocysteine methyltransferase was also used to enhance SAM regeneration; when the whole-cell catalytic methylation reaction is carried out with betaine as the methyl source, the methyl supply capacity and efficiency of this strain can be effectively improved [47]. In this study, we also examined the effects of SAM regeneration catalyzed by halide methyltransferase on SA synthesis, the SA yield (data not shown) was similar to that obtained when SAM was directly provided, indicating the feasibility of the application of the SAM regeneration system in the SA synthesis process.

Nicotinamide adenine dinucleotide phosphate (NADPH) is also a cofactor extensively involved in redox reactions in living organisms. Three intracellular pathways generate NADPH in the cell metabolic network, including the oxidative part of the pentose phosphate (PP) pathway, the tricarboxylic acid cycle, and the transhydrogenase system [48]. Many studies have focused on modifying these pathways to regulate intracellular NADPH levels. In the Pentose phosphate pathway, the genes *zwf* and *gnd* encode glucose-6-phosphate dehydrogenase and 6-phosphogluconate dehydrogenase, respectively. Overexpression of *zwf* increased NADPH levels threefold more than did *gnd* overexpression [49]. In this work, when resting cells were utilized to synthesize SA, the coupled NADPH regeneration system increased the titer of SA from 14 μM (2.7 mg/L) to 133 μM (26.2 mg/L) (Fig. 6), indicating that NADPH is a crucial cofactor for SA synthesis.

The shikimate pathway in cells provides precursors for the synthesis of aromatic amino acids and other aromatic compounds. In this work, the genes *trpED, pheA*, or *tyrA* were deleted to block the conversion of chorismate to aromatic amino acids. The bioconversion of SHA to SA catalyzed by resting whole cells reached 109 μM (21.6 mg/L). However, aromatic amino acids are important substances for cellular life, and eliminating their synthetic pathway may lead to limited cell growth and metabolism; therefore, these substances need to be provided in the culture medium.

In the final reaction solution, a significant amount of VA was detected, with a concentration even higher than that of SA. This is likely due to the fact that 3,4-DHBA, an intermediate in the bioproduction of SA from SHA, was also a substrate for the methylation reaction catalyzed by *DesA*OMT (Fig. 1). As a result, the production of VA was inevitable. Although PobA^**^ catalyzed the conversion of 3,4-DHBA to GA, its native substrate was actually 4-HBA, this could potentially limit the synthesis of GA and lead to the accumulation of 3,4-DHBA, resulting in a higher concentration of VA compared to SA in the reaction mixture. To address this issue, it is speculated that increasing the expression of PobA^**^ or modifying it to improve its affinity for 3,4-DHBA would facilitate the conversion of 3,4-DHBA to GA and ultimately lead to a higher concentration of SA. Another factor that may contribute to the higher concentration of VA was the limited availability of reducing power in the chassis cells, which was essential for the reaction catalyzed by PobA^**^. Specifically, the conversion of one molecule of 4HBA to 3,4-DHBA requires one molecule of NADPH, and the conversion of 3,4-DHBA to GA requires another molecule of NADPH. Due to the limited supply of NADPH in the cell, it may be challenging to fully convert 3,4-DHBA, resulting in increased VA production. This is supported by the results that SA production was enhanced by the addition of NADPH regeneration mixture (Fig. 6). Therefore, modification of the pentose phosphate pathway of the chassis strain to increase NADPH supply may improve SA synthesis. In addition, engineering *DesA*OMT to enhance the catalytic specificity to 3,4-DHBA might be another strategy to transform more 3,4-DHBA to SA.

Effective separation and purification techniques are crucial for the development of a complete SA biomanufacturing process. Various methods have been described for extracting SA from liquid or solid materials Among these methods, adsorption techniques have shown promise for recovering phenolic chemicals from different sources [50]. In particular, the nonpolar resin SP700 has been extensively studied for its ability to adsorb VA and SA. Within 5 min, over 83% of each phenolic acid can be easily eluted using a solution of 90% ethanol and 10% water (v/v). This results in a concentrated solution containing 5.8–11.7 g/L [51]. Liquid–liquid extraction is another effective method for extracting SA. Ionic liquids, acting as cationic hydrotrope, can increase the solubility of SA in an aqueous solution by up to 84 times compared to water. Through optimization of operational extraction conditions and the reuse solvent and biomass, the extraction yields from Rocha pear peels were found to be 2.04 to 2.22 wt% [52]. Additionally, centrifugal partition chromatography has been shown to be a useful method for the separation of five lignin-derived monomers (vanillin, vanillic acid, syringaldehyde, acetovanillone, and *p*-hydroxybenzaldehyde) with the assistance of a polyethylene glycol/sodium polyacrylate aqueous biphasic system (ABS). The system pH was found to be a crucial factor in the separation of these compounds, and the use of a buffered ABS at pH 12 in centrifugal partition chromatography allowed for selective separation of VA from the other lignin monomers [53]. In the future, the extraction of SA and VA from the biotransformation reaction solutions will be performed.

## Conclusion

SA is a high-value natural compound with various biological activities. This study developed a biosynthesis process for SA production from SHA. *DesA*OMT was identified to catalyze the methyl transfer of the meta-hydroxyl groups of catechol analogs. Then, a multienzyme cascade reaction catalyzed by AroL, UbiC, PobA^**^, and *DesA*OMT was constructed for the bioconversion of SA. Furthermore, the metabolic network of engineered *E. coli* was modified by blocking the branch pathways, and the NADPH supply in the reaction system was optimized, resulting in 133 μM (26.2 mg/L) SA production. This work provides a green route for SA synthesis.

### Supplementary Information

Supplementary Material 1.

## Data Availability

The datasets used and analyzed during the current study are available from the corresponding author upon reasonable request.

## Abbreviations

SA: Syringic acid
SAM: *S*-Adenosyl methionine
AroL: Shikimate kinase
UbiC: Chorismate lyase
PobA^**^: *p*-Hydroxybenzoate hydroxylase mutant
PNPs: Plant natural products
PCA (3,4-DHBA): Protocatechuic acid (3,4-Dihydroxybenzoic acid)
GA: Gallic acid
DHS: 3-Dehydroshikimic acid
OMT: *O*-Methyltransferase
SHA: Shikimic acid
COMT: Catechol-*O*-methyltransferase
LB: Lysogeny broth
IPTG: Isopropyl-β-d-thiogalactoside
PBS: Phosphate-buffered saline
HPLC: High-performance liquid chromatography
3MGA: 3-*O*-Methyl gallic acid
4MGA: 4-*O*-Methyl gallic acid
3,4-DMGA: 5-Hydroxy-3,4-dioxymethylbenzoic acid
SAH: *S*-Adenosylhomocysteine
VA: Vanillic acid
NADPH: Nicotinamide adenine dinucleotide phosphate
ABS: Aqueous biphasic system
