# Supplementary material for: Engineering cascade biocatalysis in whole cells for syringic acid bioproduction
Source: Microb Cell Fact. 2024 Jun 1;23:162. doi: 10.1186/s12934-024-02441-x (PMC11143566; doi:10.1186/s12934-024-02441-x)
Supplement: Supplementary file 1 — Supplementary Material 1. [file 12934_2024_2441_MOESM1_ESM.docx]

**Supplementary materials for**

**Engineering** **cascade biocatalysis in whole cells for syringic acid bioproduction**

Xin Liu, Yi An, Haijun Gao^*^

School of Life Science, Beijing Institute of Technology, Beijing 100081, China

* Corresponding author at: School of Life Science, Beijing Institute of Technology, Beijing 100081, China

*E-mail address*: [hj_gao@bit.edu.cn](mailto:hj_gao@bit.edu.cn) (H. Gao)

**TABLE OF CONTENTS**

**Supplementary methods**

Method S1. Analytical methods for assessing other phenolic compounds and corresponding O-methyl compounds

Method S2. Molecular docking

**Supplementary Tables**

Table S1. The plasmids used in this work

Table S2. The strains used in this work

Table S3. The primers used in this work

Table S4. Library of O-Methyltransferase tested in this study

**Supplementary Figures**

Figure S1. Effect of Mg^2+^(a), syringic acid(b), glucose(c), CTAB(d), and lysozyme(e) on the whole-cell catalytic synthesis of syringic acid

Figure S2. The interaction between DesAOMT and the substrate GA

**Supplementary Discussion**

**Supplementary methods**

## Method S1. Analytic methods for assessing other phenolic compounds and corresponding O-methyl compounds

In addition to SA and the intermediates produced in cascade biocatalysis, the other phenolic compounds and corresponding O-methyl compounds involved in this study were also analyzed using high-performance liquid chromatography (HPLC). The samples were centrifuged at 12,000 RPM for 2 min, after which the supernatant was collected, filtered through 0.22 μm filters, and subsequently injected into an HPLC system (Agilent 1260 Infinity II, USA) equipped with a GL Science InertSustain C-18 column (4.6×250 mm). Unless otherwise stated, the mobile phase was used at a flow rate of 0.8 mL/min. The temperature of the column was maintained at 32°C throughout the analysis. A 10 μL portion of each sample was injected for analysis using an automated injector. An ultraviolet detector was used to measure the samples. The other parameters for the specific chemicals used were as follows:

Gallic acid (GA) and its O-methyl derivatives: The mobile phase was a mixture of water containing 0.1% acetic acid and methanol (80:20, V/V), and the ultraviolet detection wavelength was set at 275 nm.

Protocatechuic acid (3,4-DHBA) and its O-methyl derivatives: A binary gradient elution of water containing 0.1% acetic acid (A) and methanol (B) was used to separate the chemicals in the samples: 0 to 25 min 70% solvent A and 30% solvent B; 25 to 30 min solvent A from 70% to 50%, solvent B from 30% to 50%, then a 10 minutes hold; 40 to 41 min solvent A from 50% to 70%, solvent B from 50% to 30%, then a hold to 50 min. The ultraviolet detection wavelength was set at 275 nm.

Caffeic acid and its O-methyl derivatives: The mobile phase was a mixture of water containing 0.1% acetic acid and methanol (55:45, V/V), and the ultraviolet detection wavelength was set at 310 nm.

3,5-Dihydroxybenzoic acid (3,5-DHBA), 2-hydroxybenzoic acid (2-HBA), 3-hydroxybenzoic acid (3-HBA), 4-hydroxybenzoic acid (4-HBA), and their O-methyl derivatives: The mobile phase was a mixture of water containing 0.1% acetic acid and methanol (55:45, V/V), and the ultraviolet detection wavelength was set at 275 nm.

2,5-Dihydroxybenzoic acid (2,5-DHBA), 2,6-dihydroxybenzoic acid (2,6-DHBA), and its O-methyl derivatives: The mobile phase was a mixture of water containing 0.1% acetic acid and methanol (65:35, V/V), and the ultraviolet detection wavelength was set at 240 nm.

2,4-Dihydroxybenzoic acid (2,4-DHBA) and its O-methyl derivatives: The mobile phase was a mixture of water containing 0.1% acetic acid and methanol (65:35, V/V), and the ultraviolet detection wavelength was set at 275 nm.

## Method S2. Molecular docking

The *DesA*OMT protein structure (PDB: 8C9V) was downloaded from the RCSB PDB database (https://www.rcsb.org/), and the 3D structure of the substrate GA (ligand, PubChem CID: 370) was downloaded from the NCBI database (https://pubchem.ncbi.nlm.nih.gov/). Discovery Studio 4.1 was used to preprocess proteins and ligands, and perform molecular docking. The Prepare Protein module was used to optimize the structure of the protein molecule, the Prepare Ligands module was used to process the small molecule, and the Define and Edit Binding Site module was used to define the possible binding sites in the receptor by finding the cavity. The CDOCKER module was used for molecular docking, and the Ligand Interactions module was used for protein and substrate docking analysis.

**Supplementary Tables**

Table S1. The strains used in this work

| **Strains** | **Characteristics** | **Sources or references** |
| --- | --- | --- |
| *E. coli* BL21(DE3) | F^–^*ompT* *hsdS_B_* (*r_B_*^–^ *m_B_*^–^) *gal dcm* (DE3) | Lab stock |
| *E. coli* MG1655 | F^–^ λ^–^ *ilvG*^–^ *rfb*–50 *rph*–1 | Lab stock |
| *E. coli* DH5α | F^–^ *φ80lacZΔ*M15 Δ(*lacZYA*-*argF*)U169 *rec*A1 *rel*A1 *end*A1 *hsd*R*17*(*r_K_*^–^ *m_K_*^+^) *pho*A *sup*E44 λ^–^*thi*-1 *gyr*A96 | Lab stock |
| LX1 | *E. coli* BL21(DE3) containing pET-28a | This work |
| LX2 | *E. coli* BL21(DE3) containing p-*StiA*OMT | This work |
| LX3 | *E. coli* BL21(DE3) containing p-*StyL*OMT | This work |
| LX4 | *E. coli* BL21(DE3) containing p-*KibP*OMT | This work |
| LX5 | *E. coli* BL21(DE3) containing p-*Syn*OMT | This work |
| LX6 | *E. coli* BL21(DE3) containing p-*RetF*OMT | This work |
| LX7 | *E. coli* BL21(DE3) containing p-*OmnB*OMT | This work |
| LX8 | *E. coli* BL21(DE3) containing p-*MyxX*OMT | This work |
| LX9 | *E. coli* BL21(DE3) containing p-*MycT*OMT | This work |
| LX10 | *E. coli* BL21(DE3) containing p-*StrA*OMT | This work |
| LX11 | *E. coli* BL21(DE3) containing p-*PhoA*OMT | This work |
| LX12 | *E. coli* BL21(DE3) containing p-*DesA*OMT | This work |
| LX13 | *E. coli* DH5α containing pTrc99a | This work |
| LX14 | *E. coli* DH5α containing pT-*DesA*OMT | This work |
| LX15 | *E. coli* DH5α containing pT-AUPD | This work |
| LX16 | *E. coli* MG1655 containing pT-AUPD | This work |
| LX17 | *E. coli* MG1655 Δ*aroE* containing pT-AUPD | This work |
| LX18 | *E. coli* MG1655 Δ*ydiB* containing pT-AUPD | This work |
| LX19 | *E. coli* MG1655 Δ*trpED* containing pT-AUPD | This work |
| LX20 | *E. coli* MG1655 Δ*pheA-tyrA* containing pT-AUPD | This work |
| LX21 | *E. coli* MG1655 Δ*aroE*Δ*ydiB* containing pT-AUPD | This work |
| LX22 | *E. coli* MG1655 Δ*aroE*Δ*ydiB*Δ*trpED* containing pT-AUPD | This work |
| LX23 | *E. coli* MG1655 Δ*aroE*Δ*ydiB*Δ*trpED*Δ*pheA-tyrA* containing pT-AUPD | This work |
| LX24 | *E. coli* MG1655 Δ*trpED*Δ*pheA-tyrA* containing pT-AUPD | This work |

Table S2. The plasmids used in this work^*^

| **Plasmids** | **Characteristics** | **Sources or references** |
| --- | --- | --- |
| pCas | *rep*A101(Ts) *P_cas_-cas9* *P_araB_-Red* *lacI^q^* *P_trc_*-sgRNA-*pMB1* *kan* | [1] |
| pET-28a | pBR322 origin, *lacI*, T7*lac*, *kan* | Lab stock |
| pTargetF | *pMB1*, *aadA*, sgRNA, *spe* | [1] |
| pTrc99a | pBR322 ori, *trc* promoter, Amp resistant | [2] |
| pE-*DesA*OMT | pET-28a harboring *DesA*OMT gene | This work |
| pE-*KibP*OMT | pET-28a harboring *KibP*OMT gene | This work |
| pE-*MyxX*OMT | pET-28a harboring *MyxX*OMT gene | This work |
| pE-*MycT*OMT | pET-28a harboring *MycT*OMT gene | This work |
| pE-*OmnB*OMT | pET-28a harboring *OmnB*OMT gene | This work |
| pE-*PhoA*OMT | pET-28a harboring *PhoA*OMT gene | This work |
| pE-*RetF*OMT | pET-28a harboring *RetF*OMT gene | This work |
| pE-*StiA*OMT | pET-28a harboring *StiA*OMT gene | This work |
| pE-*StyL*OMT | pET-28a harboring *StyL*OMT gene | This work |
| pE-*Syn*OMT | pET-28a harboring *Syn*OMT gene | This work |
| pE-*StrA*OMT | pET-28a harboring *StrA*OMT gene | This work |
| pT-AUPD | pTrc99a harboring *aroL*, *ubiC*, *pobA*^**^, and *DesA*OMT gene | This work |
| pTargetF-*aroE* | *pMB1*, *aadA*, sgRNA-*aroE* | This work |
| pTargetF-*trpED* | *pMB1*, *aadA*, sgRNA-*trpED* | This work |
| pTargetF-*pheA*-*tyrA* | *pMB1*, *aadA* sgRNA-*pheA*-*tyrA* | This work |
| pTargetF-*ydiB* | *pMB1*, *aadA*, sgRNA-*ydiB* | This work |

^*^ *kan*, kanamycin resistance gene; *spe*, spectinomycin resistance gene; *aadA*, spectinomycin resistance gene; *cat*, chloramphenicol resistance gene; *P_cas_-cas9*, the cas9 gene with its native promoter; *P_araB_-Red*, the Red recombination genes with an arabinose inducible promoter; *P_trc_*-sgRNA-*pMB1*, sgRNA with an N20 sequence for targeting the *pMB1* region with a *trc* promoter; the information for various O-methyltransferase (OMT) was provided in Table S4; *aroL*, shikimate kinase; *ubiC*, chorismate lyase; *pobA*^**^, *p*-hydroxybenzoate hydroxylase mutant gene from *Pseudomonas fluorescens*; sgRNA-*aroE*, sgRNA-*ydiB*, sgRNA-*trpED*, and sgRNA-*pheA*-*tyrA* sgRNA with an N20 sequence for targeting the *aroE*, *ydiB*, *trpED*, *pheA*-*tyrA* locus, respectively.

Table S3. The primers used in this work

| **Primers** | **Sequences (5’-3’)** | **Description** |
| --- | --- | --- |
| PTRC99C-F | Ttgcgccgacatcataac | Verification |
| PTRC99C-R | ctgcgttctgatttaatctg | Verification |
| T7 | taatacgactcactatagg | Verification |
| T7-TER | gctagttattgctcagcgg | Verification |
| StiAOMT-F | aagaaggagatataccatgaatgaaaaagtcatggcggtg | Construction of pET-*StiA*OMT |
| StiAOMT-R | cggagctcgaattcgtcaggtgggctcgaaccg | Construction of pET-*StiA*OMT |
| StyLOMT-F | aagaaggagatataccatggaagatctaaataaagataaat | Construction of pET-*StyL*OMT |
| StyLOMT-R | cggagctcgaattcgtcatttcttctttactattgtcaacccat | Construction of pET-*StyL*OMT |
| KibPOMT-F | aagaaggagatataccgtgacacccgaatggccg | Construction of pET-*KibP*OMT |
| KibPOMT-R | cggagctcgaattcgtcaggctgtcttcgccgc | Construction of pET-*KibP*OMT |
| slr0095-F | ttgtttaactttaagaaggagatataccatgggtaagggcatcaccg | Construction of pET-*Syn*OMT |
| slr0095-R | gtcgacggagctcgaattcgctattttttgagtgccaaagtcatg | Construction of pET-*Syn*OMT |
| RetFOMT-F | aagaaggagatataccatgaatagggcgacgtttataagg | Construction of pET-*RetF*OMT |
| RetFOMT-R | cggagctcgaattcgttacaattttcgaacaaacgtaac | Construction of pET-*RetF*OMT |
| OmnBOMT-F | aagaaggagatataccatgaatccaattcatcatgaactca | Construction of pET-*OmnB*OMT |
| OmnBOMT-R | cggagctcgaattcgtcatttttttcgcgccagtgtaa | Construction of pET-*OmnB*OMT |
| SafC-F | ttgtttaactttaagaaggagatataccatgatccaccacgtcgaattg | Construction of pET-*MyxX*OMT |
| SafC-R | gtcgacggagctcgaattcgctagcgcttgcgagcgag | Construction of pET-*MyxX*OMT |
| Rv0187-F | ttgtttaactttaagaaggagatataccatggaccagcaacccaacc | Construction of pET-*MycT*OMT |
| Rv0187-R | gtcgacggagctcgaattcgctaccgcaccaaagcgagg | Construction of pET-*MycT*OMT |
| StrAOMT-F | aagaaggagatataccatgagcgagtcgcaacagct | Construction of pET-*StrA*OMT |
| StrAOMT-R | cggagctcgaattcgctacggcagcacgcgg | Construction of pET-*StrA*OMT |
| PhoAOMT-F | aagaaggagatataccatgacaaaaaagacactgggattgga | Construction of pET-*PhoA*OMT-F |
| PhoAOMT-R | cggagctcgaattcgtcaacgtttccgtgccaaagttaaa | Construction of pET-*PhoA*OMT-F |
| DesAOMT-F | aagaaggagatataccatgaataaagaattgcaccagcttct | Construction of pET-*DesA*OMT |
| DesAOMT-R | cggagctcgaattcgtcatgcagagcgggtggc | Construction of pET-*DesA*OMT |
| pTD-F | caggaaacagaccatggaattcatgaataaagaattgcaccagc | Construction of pT-*DesA*OMT |
| pTD-R | aggtcgactctagaggatcctcatgcagagcgggtg | Construction of pT-*DesA*OMT |
| aroL-F | acacaggaaacagaccatggaattcatgacacaacctctttttctgat | Construction of pT-AUPD |
| aroL-R | tatttctcctctttctctagatcaacaattgatcgtctgtgc | Construction of pT-AUPD |
| Ubic-F | tctagagaaagaggagaaatactagatgtcacaccccgcg | Construction of pT-AUPD |
| Ubic-R | gtcgactctagaggatccttagtacaacggtgacgcc | Construction of pT-AUPD |
| pobA_**_-F | taaggatcctctagagtcgactctagagttcacacaggaaacc | Construction of pT-AUPD |
| pobA*^**^*-R | ctcctctttaatctctagactactcgatttcctcgtagg | Construction of pT-AUPD |
| RBSDes-F | tctagagattaaagaggagaaatactagatgaataaagaattgcacc | Construction of pT-AUPD |
| RBSDes-R | gccaaaacagccaagctttcatgcagagcgggt | Construction of pT-AUPD |
| aroE-1 | ccgagtcggtgctttttttgaattcAACGGAAGCCGTTTTCG | Construction of donor DNA for *aro*E disruption |
| aroE-2 | GATGGCCTGATTATGTTACCCCTGTCGAAAC | Construction of donor DNA for *aro*E disruption |
| aroE-3 | GGGGTAACATAATCAGGCCATCCAGTTTC | Construction of donor DNA for *aro*E disruption |
| aroE-4 | agggataacagggtaatagatctaagcttCCACGGCTGCACCATTG | Construction of donor DNA for *aro*E disruption |
| ydiB-1 | ccgagtcggtgctttttttgaattcATGGGCTCGTTGGTCTGT | Construction of donor DNA for ydiB disruption |
| ydiB-2 | CGGTCAGCCTGAATTAACTCCTTTAACCTCTATGCT | Construction of donor DNA for ydiB disruption |
| ydiB-3 | GGAGTTAATTCAGGCTGACCGCGTGC | Construction of donor DNA for ydiB disruption |
| ydiB-4 | ggataacagggtaatagatctaagcttCGGGCAATGATTTCTTCG | Construction of donor DNA for ydiB disruption |
| trpED-1 | gagtcggtgctttttttgaattcAGGAACTCACACATTAGCG | Construction of donor DNA for trpED disruption |
| trpED-2 | CGGTTTGCATCATTGTTATTCTCTAATTTTGTTCAA | Construction of donor DNA for trpED disruption |
| trpED-3 | TAGAGAATAACAATGATGCAAACCGTTTTAGCGA | Construction of donor DNA for trpED disruption |
| trpED-4 | agggataacagggtaatagatctaagcttTTACTGACTTCGGTCAGCAC | Construction of donor DNA for trpED disruption |
| pheA-tyrA-1 | gagtcggtgctttttttgaattcAAACACATCTGATTAATCCACA | Construction of donor DNA for pheA-tyrA disruption |
| pheA-tyrA-2 | AAGAGGTTTATTAGTGTTGCCTTTTTGTTATCAATA | Construction of donor DNA for pheA-tyrA disruption |
| pheA-tyrA-3 | AAAGGCAACACTAATAAACCTCTTAAGCCACGC | Construction of donor DNA for pheA-tyrA disruption |
| pheA-tyrA-4 | ataacagggtaatagatctaagcttGGCAACAGCAATTAACGCTA | Construction of donor DNA for pheA-tyrA disruption |
| pTargetF.fk-R | ACTAGTATTATACCTAGGACTGAGCTAGCTGTCAAG | Construction of pTargetF-sgRNA |
| aroEN20-1-F | TCCTAGGTATAATACTAGTGCAGAATACGTAAACCAGGGGTTTTAGAGCTAGAAATAGC | Construction of pTargetF-*aro*E |
| ydiBN20-1-F | TCCTAGGTATAATACTAGTCTGCGTGGCTATAACACCGAGTTTTAGAGCTAGAAATAGC | Construction of pTargetF-*ydi*B |
| trpEDN20-2-F | TCCTAGGTATAATACTAGTATTGCCGGAACACGCCCACGGTTTTAGAGCTAGAAATAGC | Construction of pTargetF-*trp*ED |
| pheAtyrAN20-1-F | TCCTAGGTATAATACTAGTTGTACCGCCAGATCTGATTGGTTTTAGAGCTAGAAATAGC | Construction of pTargetF-*phe*A-*tyr*A |
| aorE.700-F | CCATAGCTGGAGAATGCCCT | Verification of the deletion to *aroE* |
| aorE.700-R | GGTGACGTTCGTCTGGCTGA | Verification of the deletion to *aro*E |
| ydiB.700-F | GCCAGCGTAACTAATGAATT | Verification of the deletion to *ydi*B |
| ydiB.700-R | AGCCAAATACTTCACCAGCC | Verification of the deletion to *ydi*B |
| trpED.700-F | TTCGGCAATCAGCTGCGC | Verification of the deletion to *trp*ED |
| trpED.700-R | AACCGTTAGCGAAGTGGC | Verification of the deletion to *trp*ED |
| pheAtyrA.700-F | ACGGTATGCTGAATTCACCA | Verification of the deletion to *phe*A-*tyr*A |
| pheAtyrA.700-R | CGTAAATTGCTGCTTGAGCT | Verification of the deletion to *phe*A-*tyr*A |

Table S4. Library of O-Methyltransferase tested in this study

| Protein names | Uniprot entry name | Sources |
| --- | --- | --- |
| *StiA*OMT | E3FEM3_STIAD | *Stigmatella aurantiaca* (DW4/3-1) |
| *StyL*OMT | A0A078AUZ0_STYLE | *Stylonychia lemnae* |
| *KibP*OMT | A0A0N9HPV5_9PSEU | *Kibdelosporangium phytohabitans* |
| *Syn*OMT | Q55813_SYNY3 | *Synechocystis sp.* (PCC 6803/Kazusa) |
| *RetF*OMT | X6M5Z7_RETFI | *Reticulomyxa filosa* |
| *OmnB*OMT | A0A1G1JPP6_9BACT | *Omnitrophica bacterium* GWA2_52_8 |
| *MycT*OMT | CAMT_MYCTU | *Mycobacterium tuberculosis* (ATCC 25618/H37Rv) |
| *MyxX*OMT | Q50859_MYXXA | *Myxococcus xanthus* |
| *StrA*OMT | Q82B68_STRAW | *Streptomyces avermitilis* (ATCC 31267/DSM 46492) |
| *PhoA*OMT | A0A1U7IJN5_9CYAN | *Phormidium ambiguum* IAM M-71 |
| *DesA*OMT | Q1JXV1_DESA6 | *Desulfuromonas acetoxidans* (DSM 684/11070) |

**Supplementary Figures**


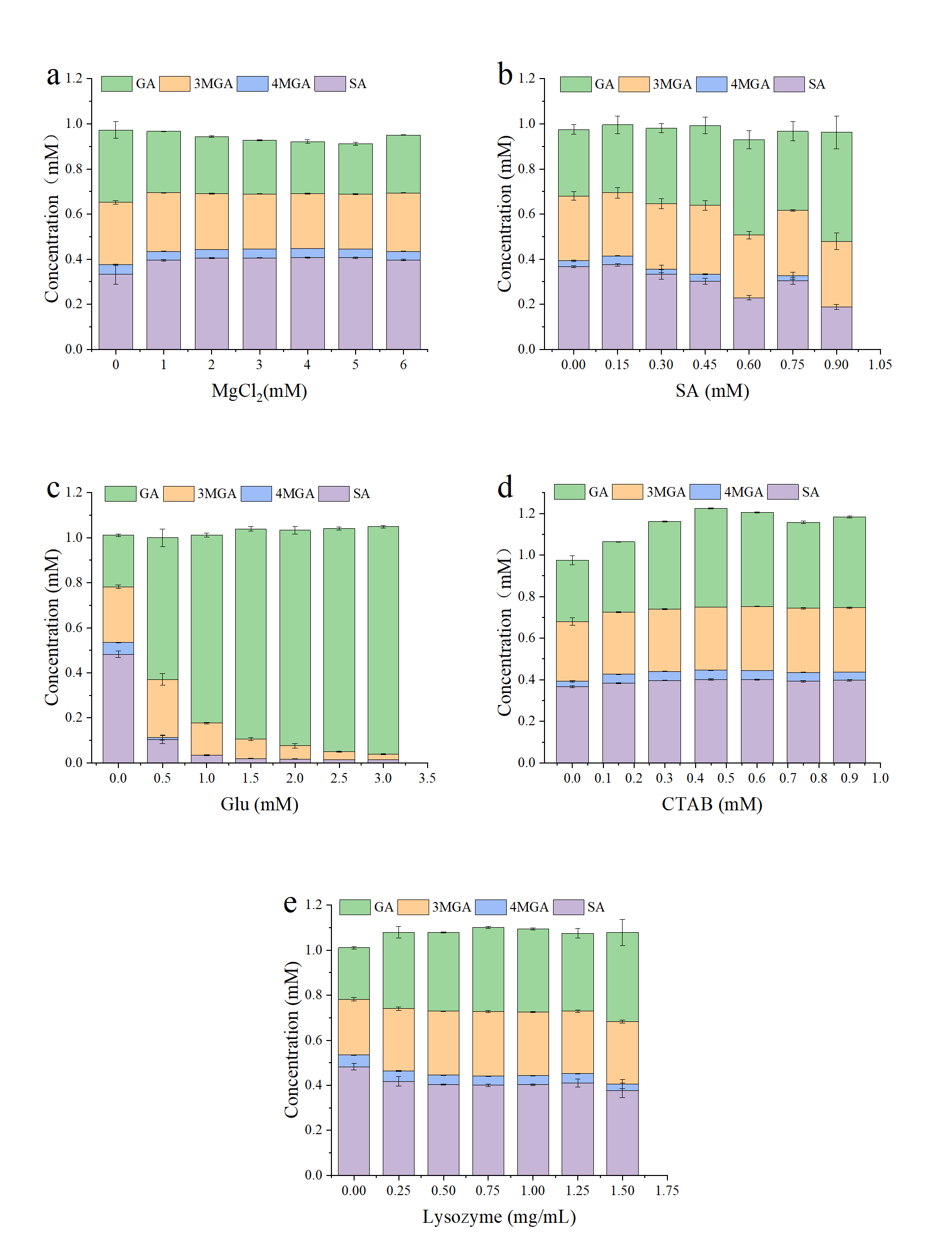


Figure S1 Effect of Mg^2+^(a), syringic acid(b), glucose(c), CTAB(d), and lysozyme(e) on the yields of syringic acid catalyzed by using resting whole cells. The *E. coli* LX12 was cultivated in shake flasks at 190 rpm and 37°C for 5 h, 0.5 mM IPTG was added to induce gene expression, and subsequent cultivation was conducted at 30°C for 18 hours. Then the cells were collected (OD_600nm_ of 9) and subjected to catalysis with 1.0 mM gallic acid for 8 hours. The error bars represent the standard deviations of the means of three independent measurements. Abbreviations: GA, gallic acid, 3MGA, 3-O-methyl gallic acid, 4MGA, 4-O-methyl gallic acid, SA, syringic acid.


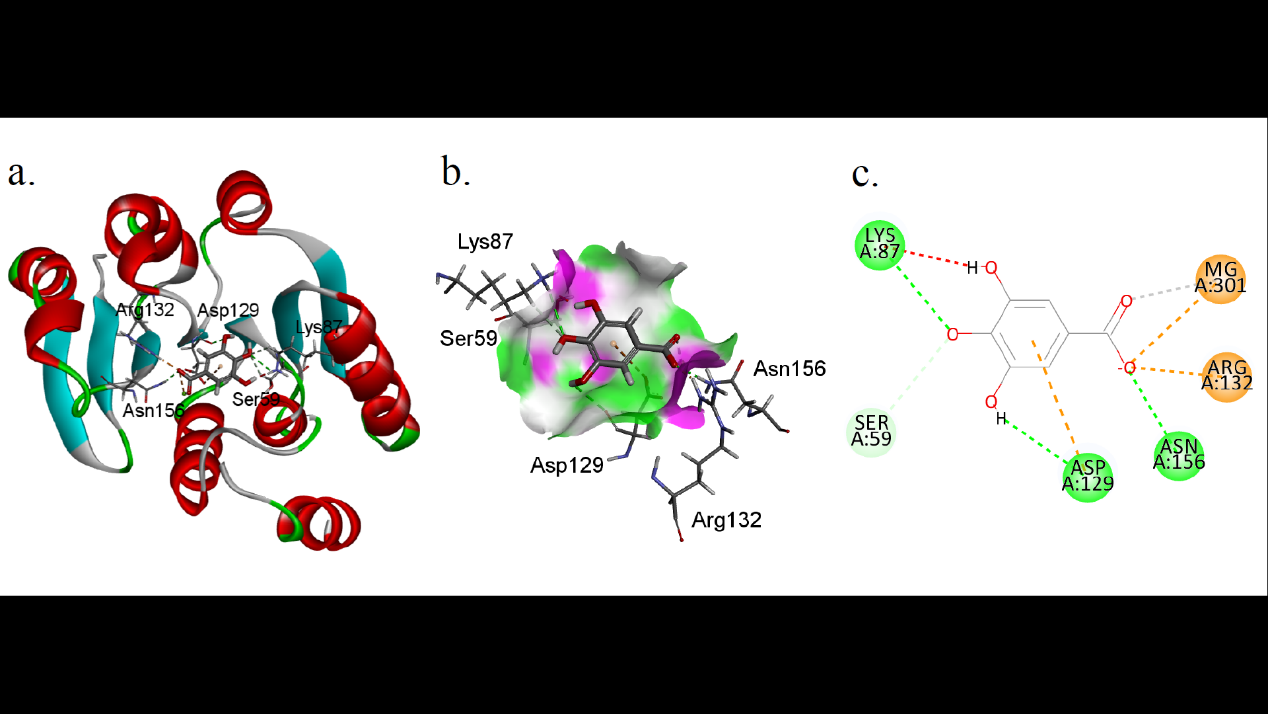


Figure S2. A diagram of the predicted interactions between the protein (*DesA*OMT) and ligand (GA). (a) Ribbon diagram of the *DesA*OMT-GA complex. The active amino acid residues and GA are shown in a ball-and-stick molecular structure. (b) H-Bonds surfaces on the active site of *DesA*OMT. The active amino acid residues and GA are shown in a stick molecular structure. (c) 2D diagram of the interaction between GA and *DesA*OMT. The colored circular shapes represent amino acid residues or metal ions. The green, yellow, and red dashed lines represent hydrogen bonds, electrostatic interaction, and donor–donor clashes, respectively.

**Supplementary Discussion**

The overall structure of DesAOMT (PDB: 8C9V) is a dimer formed by two subunits composed of 195 amino acids. The core structure of *DesA*OMT is a Rossmann fold containing 7 β-sheets and 7 α-helixes. Unlike other reported COMTs, *DesA*OMT is devoid of the N-terminal α-helix that is implicated in dimerization[3-5]. The interaction between *DesA*OMT and Gallic acid (GA) was studied using Discovery Studio. Our docking results showed that the carboxyl group of GA interacted with two amino acid residues of *DesA*OMT, Arg132, which formed electrostatic interactions, and Asn156, which formed conventional hydrogen bonds with a length of 2.12 Å. These two interactions may immobilize the substrate molecule. The para hydroxyl group of GA interacted with two amino acid residues, Lys87 formed a strong hydrogen bond with a length of 2.83 Å, and Ser59 formed a weak intermolecular hydrogen bond with a length of 2.96 Å. The ortho hydroxyl group of GA and the amino acid residue Asp129 formed a strong hydrogen bond with a length of 2.90 Å. In addition, Asp129 had a weak interaction force with the aromatic ring and may contribute to the fixation of substrate molecules. These docking results suggested that Lys87, Asp129, and Asn156 may contribute more to the catalytic activity of the enzyme. The surrounding hydrophobic amino acids further stabilized the *DesA*OMT-GA complex and improved its catalytic activity and thermostability.

References

1. Jiang Y, Chen B, Duan C, Sun B, Yang J, Yang S. Multigene editing in the *Escherichia coli* genome via the CRISPR-Cas9 system. Appl Environ Microbiol. 2015;81:2506-14.

2. Amann E, Ochs B, Abel KJ. Tightly regulated tac promoter vectors useful for the expression of unfused and fused proteins in Escherichia coli. Gene. 1988;69:301-15.

3. Lee SH, Kim B, Kim K-J. Crystal structure and regiospecificity of catechol O-methyltransferase from *Niastella koreensis*. J Agric Food Chem. 2021;69:2531-8.

4. Kopycki JG, Stubbs MT, Brandt W, Hagemann M, Porzel A, Schmidt J, et al. Functional and structural characterization of a cation-dependent O-methyltransferase from the cyanobacterium *Synechocystis* sp strain PCC 6803. J Biol Chem. 2008;283:20888-96.

5. Lee S, Kang J, Kim J. Structural and biochemical characterization of Rv0187, an O-methyltransferase from *Mycobacterium tuberculosis*. Sci Rep. 2019;9:8059.
